# Hydrogeomorphological Assessment and Prioritized Modelling of Groundwater Scarcity in the Kangsabati River Basin Using Multiattribute Decision‐Making (MADM) Approaches

**DOI:** 10.1155/sci5/9965060

**Published:** 2026-10-08

**Authors:** Sanjay Saha, Menuka Gautam, Jayanta Das

**Affiliations:** ^1^ Department of Geography, Kaliachak College, Sultanganj Kaliachak, Malda Pin-732201, India; ^2^ Central Department of Geology Tribhuvan University Kirtipur Kathmandu Post Box: 44600 Nepal tribhuvan-university.edu.np; ^3^ Department of Geology Birendra Multiple Campus Tribhuvan University Bharatpur Chitwan Nepal tribhuvan-university.edu.np; ^4^ Department of Geography Rampurhat College Rampurhat Birbhum 731224 India

**Keywords:** groundwater scarcity, hydrogeomorphology, Kangsabati River Basin, multicriteria decision-making analysis, stream length-gradient (SL) index

## Abstract

Groundwater resource depletion constitutes a major global environmental challenge, requiring robust quantitative frameworks to optimize localized water sustainability initiatives. This study examines the hydrogeomorphological characteristics of the Kangsabati River Basin to assess groundwater scarcity and identify priority areas for management. Total 47 morphometric indicators were analysed to understand their influence on groundwater conditions. The basin shows a well‐developed but uneven drainage network, reflecting marked differences in infiltration potential and groundwater availability across subwatersheds. To rank the subwatersheds based on the severity of groundwater scarcity, six multiattribute decision‐making (MADM) methods were applied. The analysis incorporated widely used techniques, including the Technique for Order of Preference by Similarity to Ideal Solution (TOPSIS), Vlsekriterijumska Optimizacija I Kompromisno Resenje (VIKOR), simple additive weighting (SAW), composite compromise solution (CoCoSo), multiobjective optimization by ratio analysis (MOORA) and evaluation based on distance from average solution (EDAS). The combined assessment highlights the complexity of comparing groundwater stress across a geomorphologically diverse basin while also demonstrating the value of MADM approaches for producing consistent and transparent prioritisation. The findings reveal significant spatial variability in groundwater conditions, with several subwatersheds such as 1, 2, 4, 5, 6, 14, 15 and 18 showing clear signs of scarcity. These results highlight the need for targeted recharge measures, improved water conservation strategies and more effective watershed‐level planning. This study provides a structured framework for assessing groundwater scarcity and supports informed decision‐making for sustainable water resource management in the Kangsabati River Basin.

## 1. Introduction

Anthropogenic groundwater depletion constitutes a pervasive global environmental threat, requiring high‐resolution quantitative frameworks capable of optimizing localized, catchment‐scale sustainability initiatives [1]. At the initial stage of civilization, people depend on the available surface water reservoirs to fulfil their water requirements [2]. With the progress of society, technologically advanced people try to search for substitute sources of water other than surface water through the discoveries of innovative technologies [3]. Since the discoveries of sophisticated technologies, people have started extracting groundwater by several methods; this is the beginning of the depletion of groundwater, which leads to chronological environmental degradation [4–6]. The heterogeneous geoenvironmental composition of the earth’s surface gives spatial constrain to groundwater extraction worldwide. Thus, it is necessary to understand groundwater storage status before extracting groundwater at any place [7, 8]. In the modern technologically advanced era, groundwater can be extracted with unprecedented efficiency via high‐capacity mechanized pumping wells. However, the absence of stringent regulatory boundaries often translates this enhanced capacity into localized over‐exploitation. Such unregulated and irrational abstraction outpaces natural aquifer replenishment, leading to a severe chronological decline in groundwater storage that threatens human civilization. Owing to the groundwater crisis, overall physical and biological functions are being disrupted, hampering the delicate balance of the water cycle [9]. Groundwater paucity is causing problems in sustaining the livelihoods of communities, which is necessitating the assessment of spatial groundwater potentiality by experts, including hydrologists, environmentalists, geomorphologists and decision‐makers [10–12].

The groundwater storage potential defines the capacity of water confined within an aquifer [13]. The groundwater potential is linked with the geological, climatic and hydrological aspects of a region [14]. Thus, for determining the groundwater potential of river basin, it is necessary to understand the rock porosity, permeability, open space in rock and soil, intensity and amount of rainfall, infiltration rate and runoff [15–17]. A systematic study of geological, hydrological, climatic and geomorphic features is helpful for assessing the groundwater potentiality of a region [5, 18, 19].

Geomorphologists and hydrologists eagerly focus on methodologically microwatershed prioritization while assessing the groundwater potentiation in the drainage basin as a fundamental hydrogeomorphological unit [20]. To formulate strategies for effective groundwater management in drainage basins, comprehension of the hydrogeomorphic features of the basin is essential [21, 22]. Several engineering mechanical methods are used for measuring groundwater status in a drainage basin, but these are costly [23]. Alternatively, geomorphologists and hydrologists search for theoretical approaches while estimating groundwater potentiality, and these are commonly used for low‐cost methods [24]. In this connection, morphometric analysis, a quantitative technique that measures the surface configuration of the earth, is accepted as an essential method for assessing groundwater storage conditions in a river basin [21, 25, 26].

The river basin’s geometrical arrangement, morphological expression and hydrological characteristics can be understood by morphometric analysis, which includes linear, areal and relief aspects [27]. The region’s expression morphology results from the interaction of different physical sets and processes like tectonic activities, lithology, geologic structure, climate and geomorphic processes of the associated region [28]. The exposed geomorphic features of a region convey their influencing factors and give an insight into the underground storage and potentiality [29, 30]. Understanding the morphological characteristics of a river basin helps to explore its hydrological characteristics. This can be achieved through accurate and validated morphometric analysis [31]. For this reason, a geomorphologist tries to validate the accuracy of derived results of morphometric analysis through different statistical tests and mathematical models. A precise and structurally rigorous quantitative analysis of a drainage basin’s morphometry assists in assessing its underground storage and potentiality [32]. Contemporarily, researchers are keenly interested in applying multicriteria decision‐making (MCDM) methods to prioritize subbasins based on the availability or paucity of groundwater within a large river basin through the analysis of its morphometric characteristics [33–35]. Inferring the groundwater status of a region with the help of morphometric parameters through MCDM necessitates validation with the real‐world situation [36, 37].

Crucially, a holistic paradigm of water security dictates that quantitative groundwater availability mapping must coexist with qualitative preservation. Globally, highly productive aquifer networks frequently undergo rapid chemical degeneration or anthropogenically driven contamination, rendering water unsafe despite physical abundance. This critical nexus is observed in various geomorphological settings across the world, including heavy metal and mineral contamination in the unconfined systems of Algeria and Nigeria, industrial‐agricultural runoff degradation in parts of India, severe salinization dynamics in the coastal and interior belts of Ghana and rapid chemical depletion alongside quality degradation in hyperarid blocks of Saudi Arabia. Consequently, modelling spatial quantitative paucity serves as the foundational structural sorting layer prior to deploying targeted pollution remediation frameworks.

This study has attempted to prioritize subwatersheds based on the groundwater scarcity in the Kangsabati River Basin. The status of groundwater scarcity has been assessed based on the relevant morphometric parameters that influence the status of groundwater by scrutinizing the results through MCDM methods, such as TOPSIS, VIKOR, SAW, CoCoSo, MOORA and EDAS, and validation against the real‐world situation. This study aims to explore the status of groundwater scarcity in the Kangsabati River Basin. This work will assist to chalk out effective strategies to mitigate the groundwater scarcity in the Kangsabati River Basin. An integrative approach has been used in this study, merging the domains of geomorphology, hydrology and decision‐making sciences to excavate the vital issue of groundwater paucity, which is the uniqueness of this research work. Such an innovative technique helps the subbasin prioritization based on groundwater scarcity, signifying the study’s nobility. The derived results from each selected method of MCDM are validated with real‐world situations, demonstrating the practical field applicability and empirical validity of the model output. This research work could contribute to a better understanding of groundwater dynamics and give practical insights for effective, sustainable and efficient management of groundwater resources, especially in river basins. The specific objectives of this investigation are to (i) quantify 47 distinct linear, areal and relief morphometric features across 18 subwatersheds using high‐resolution SRTM telemetry; (ii) run a comparative priority simulation of groundwater scarcity using six autonomous MADM architectures and (iii) perform cross‐sectional and stream length‐gradient (SL) indexing to confirm structural controls on water stress distribution.

## 2. Study Area

The Kangsabati River performs a significant role in livelihoods and is located in the Western Plateau region of West Bengal, India. The river is named as Kasai and Cossve by the native people in the basin [38, 39]. The river’s source is on the Chota Nagpur Plateau near Jhalda in the Purulia district. The river flows south‐eastward, passing through the districts of Purulia, Bankura, Paschim Medinipur and Purba Medinipur in West Bengal, India. The river is mingled with the Keleghai, and Keleghai mingles with the Haldi River and finically debouches in the Bay of Bengal. The total length of the river Kangsabati is 465.23 km, and its base covers a total geographical area of about 5620.61 square kilometres. The basin of Kangsabati extends from 85° 45′ E to 87° 37′ E longitudes and 21° 55′ N to 23° 25′ N latitudes (Figure 1).

**FIGURE 1:**
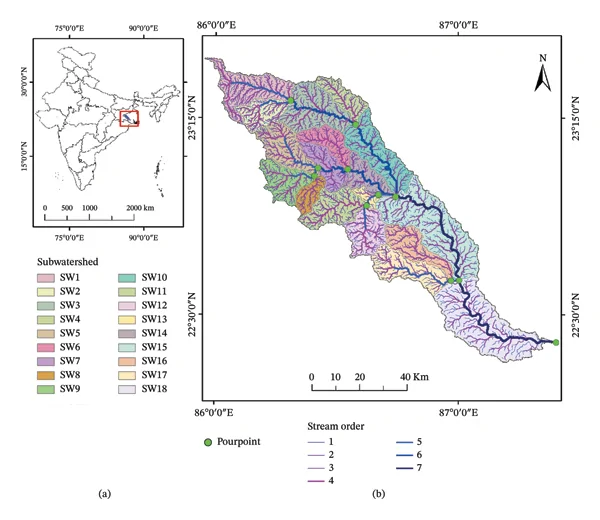
Location map of the study area and watershed delineation: (a) India and (b) Kangsabati River Basin (KRB) and stream ordering.

The geological heterogeneity of the basin reveals a tapestry of features. The upstream section of the basin is a part of the Chota Nagpur Plateau characterised by rugged terrain having a maximum elevation of about 641 m. In the upstream section, the oldest Archaean formation comprises granite and gneiss rocks. The middle segment of the basin, geologically, belongs to the formation of the Cambrian era, mainly composed of laterite. Tertiary‐quaternary formation prevails in the lower segment of the basin, which is characterised by a low elevated floodplain and is primarily composed of laterite. Extensive alluvial deposits formed the bedrock of the lower segment of the basin during the Pleistocene to Holocene epochs [30, 40]. Due to unequal denudational activities on the basin’s diversified geological structure and lithology, a dendritic drainage pattern has evolved. The basin has 4143 streams engaged in gully erosion, mainly in the southwest monsoon season due to monsoon showers. The discharge in the Kangsabati River Basin is highly fluctuating as the region is experienced with moderately wet rainy seasons and dry summer and winter seasons. The average rainfall in the basin is about 165 cm annually, but it also experiences a drought condition in the summer, resulting in a paucity of water in the basin’s drainage network. Although the basin is drought‐prone, the Kangsabati River is a lifeline for its neighbourhoods as it provides water for irrigation and domestic purposes.

The Kangsabati Reservoir Project has been initiated at the confluence of the river Kangsabati and its tributary Kumari River to manage the flood phenomena in the lower segment of the basin and meet the water needs of the basin’s dwellers [41, 42]. Thus, the groundwater status in the basin must be estimated for the well‐being of the basin’s dwellers [43].

## 3. Data Source and Methods

### 3.1. Data Source

The primary goal of this research is to prioritize sub‐basins in the Kangsabati River Basin based on the predicted groundwater paucity in the basin, which is estimated by analysing the basin’s morphometric parameters. The SRTM Digital Elevation Model (DEM) is used to extract the basin’s morphometric parameters, which are collected from the USGS Earth Explorer website (https://earthexplorer.usgs.gov/).

### 3.2. Methodology

#### 3.2.1. Methods for Morphometric Analysis

A morphometric analysis was conducted to study the basin and its subwatersheds at both macro and micro levels. Based on stream outlets, we identified eighteen subwatersheds. To understand the basin’s overall morphometric characteristics, we examined 47 morphometric variables, including linear, areal and relief aspects. Indeed 24 morphometric variables have been taken into consideration which are relevant to evaluate the groundwater conditions in the subwatersheds. The detailed methodology is described in Figure 2.

**FIGURE 2:**
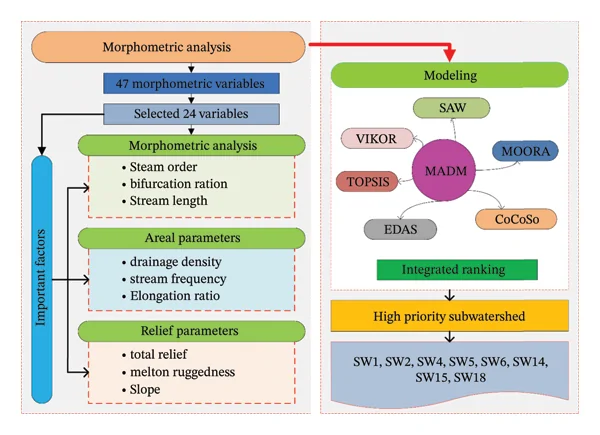
Methodological framework of groundwater scarcity in the Kangsabati River Basin.

#### 3.2.2. Methods for Multicriteria Decision‐Making (MCDA)

To prioritize subwatersheds based on the severity of groundwater scarcity, six multiattribute decision‐making (MADM) methods were used. There are several decision‐making analysis techniques available, including the Technique for Order of Preference by Similarity to Ideal Solution (TOPSIS), Vlsekriterijumska Optimizacija I Kompromisno Resenje (VIKOR), simple additive weighting (SAW), composite compromise solution (CoCoSo), multiobjective optimization by ratio analysis (MOORA) and evaluation based on distance from average solution (EDAS). The models used in this study are discussed as followed:

##### 3.2.2.1. The TOPSIS Model

Ching‐Lai Hwang and Yoon (1981) introduced the TOPSIS model to detect the most favourable alternative by estimating its closeness to the positive ideal solution (PIS) and its remoteness from the negative ideal solution (NIS) [44]. Yoon revised this model in 1987, and Hwang, Lai and Liu modified it again in 1993.

Steps in TOPSIS are as follows:

I. Describing criteria and alternatives:
(1) fdm=Nciji=1212,,…c;j=,,……,n,
 
*m* = number of criteria
 
*n* = number of attributes
 
*c* *i* *j* = performance of alternative i under criteria j
II. Framing the decision matrix (*f*_*d* *m*_):
 The decision matrix comprises preferred alternatives and decisive attributes, with beneficial and nonbeneficial criteria denoted by *b*_*f*_ and *n* *b*_*f*_, respectively.
III. Normalization of decision matrix:
 Each decisive attribute is normalized using the formula
(2) Ncij=Cij∑j=1cCij2,
 where *N*_*c* *i* *j*_ = normalized decisive attribute, *C*_*i* *j*_ = decisive attribute
IV. Weighted normalized decisive attribute:
 Weighted normalized criteria are calculated using the formula
(3) WNCij =Wi ×Ncij.
 
*W* *N*_*C* *i* *j* _ = weighted normalized criteria
V. Finding the PIS and NIS (*P*_*I* *S* _ and *N*_*I* *S* _):
 
*P*_*I* *S* _ represents the maximum normalized weighted value of beneficial attributes and the minimum normalized weighted value of nonbeneficial attributes.
 
*N*_*I* *S* _ represents the minimum normalized weighted value of beneficial attributes and the maximum normalized weighted value of nonbeneficial attributes.
VI. Operation matrix:
(4) OMp=∑IWNCij −PIS 2,
(5) OMn=∑IWNCij −NIS 2.
 Explanation: *O*_*M* *p*_ = operation matrix for PIS
 
*O*_*M* *n*_ = operation matrix for NIS
 
*I* *W* *N*_*C* *i* *j* _ = individual normalized weighted value of the attribute
 
*P*_*I* *S* _ = positive ideal solution
 
*N*_*I* *S* _ = negative ideal solution
VII. Relative closeness coefficient *R*_*C* *C*_:
(6) RCC=OMp÷OMp+OMn.
VIII. Ranking of alternatives:
 Alternatives are ranked in descending order based on the value of the relative closeness coefficient.

##### 3.2.2.2. VIKOR Method

A method for multicriteria optimization and compromise solution, which is akin to the TOPSIS model, was introduced by Opricovic and Tzeng in 1998 named The VIKOR. This model finally ranks the alternatives based on the comprehensive evaluation index (CEI) value [45–47]. The steps involved in the VIKOR method are as follows:

I. Framing of decision matrix:
(7) fdm=Nciji=1212,,…c;j=,,……,n,
 
*m* = number of criteria
 
*n* = number of attributes
 
*c* *i* *j* = performance of alternative i under criteria j
II. Normalization of decision matrix:
(8) Ncij=Cij∑j=1cCij2,
III. where *N*_*c* *i* *j*_ = normalized decisive attribute and *C*_*i* *j*_ = decisive attribute.
IV. Calculation of utility measure (Ui) and regret measure (*R*_*g* *i*_):
(9) Ui=∑i=1nWiNCijmax−NCijNCijmax−NCijmin for beneficial attribute,
(10) Ui=∑i=1nWiNCij−NCijminNCijmax−NCijmin for nonbeneficial attribute,
(11) Rgi=maximum of WiNCijmax−NCijNCijmax−NCijmin for beneficial attribute,
(12) Rgi=maximum of WiNCij−NCijminNCijmax−NCijmin For nonbeneficial attribute.
 
*W*_*i*_ = assigned weight
V. Calculation of CEI:
(13) comprehensive evaluation index CEI=VUi−SiminUimax−Uimin+1−VRgi−RgiminRgimax−Rgimin,
 
*V* = value is considered as 0.5. However, the value of V can be any value between 0 and 1
VI. Ranking of alternatives:
 Alternatives are ranked in descending order based on the CEI value.

##### 3.2.2.3. SAW Method

One of the direct methods of MCDM is the SAW method, which chooses the alternative according to the weighted score of the attributes [48, 49]. The steps of the SAW method can be summarized as follows:

I. Framing of decision matrix:
(14) fdm=Nciji=1212,,…c;j=,,……,n,
 
*m* = number of criteria
 
*n* = number of attributes
 
*c* *i* *j* = performance of alternative i under criteria j
II. Normalization of decision matrix:
(15) Ncij=CijCijmax for beneficial attribute,
(16) Ncij=CijminCij for beneficial attribute.
III. Calculation of weighted scores:
(17) WNcij=Wi×NCij.
IV. Summation of weighted scores:
(18) SWA=∑j=1nWi×NCij.
V. Ranking of alternatives:
 Alternatives are ranked in descending order based on the value of the weighted score.

##### 3.2.2.4. Combined Compromise Solution (CoCoSo) Method

An innovative model of MCDA introduced by Wątróbski and Yazdani (2019) is the CoCoSo which integrates the algorithm with SAW product model and exponential weighted product model. It enables to make decision in the combination of different strategies for choosing alternative.

The summarized steps of the CoCoSo method are as follows.

I. Framing of input decision matrix:
(19) fdm=Nciji=1212,,…c;j=,,……,n,
 
*m* = number of criteria
 
*n* = number of attributes and *c* *i* *j* = performance of alternative i under criteria j
II. Normalization of input decision matrix:
(20) Ncij=Cij−CijminCijmax−Cijmin for beneficial attribute,
(21) Ncij=Cijmax−CijCijmax−Cijmin for beneficial attribute.
III. Sum of the weighted comparability:
(22) Swi=∑j=1nWi×NCij.
IV. Power of the weighted comparability matrix:
(23) Pwi=∑j=1nCijWi.
V. Aggregation of appraisal score strategies:
(24) Aia=Swi+Pwi∑i−1mSwi+Pwi,
(25) Aib=SwiSwimin+PwiPwimin,
(26) Aic=ℷSwi+1−ℷPwiℷmaxSwi+1−ℷmaxPwi01<ℷ<.
 
*A*_*i* *a*_, *A*_*i* *b*_ and *A*_*i* *c*_ values are to be ranked separately in descending order to compare the similarity of the rank
VI. Combined aggregation of appraisal score strategy:
(27) Ai=Aia×Aib×Aic1313/+/Aia+Aib+Aic.
 
*A*_*i*_ value is to be ranked in descending order to compare the similarity of ranks as done as per the values of *A*_*i* *a*_, *A*_*i* *b*_ and *A*_*i* *c*_

##### 3.2.2.5. MOORA

Compared to more complex MCDM methods like ANP or TOPSIS, the MOORA framework requires fewer computational steps while maintaining a high degree of stability against rank reversal [50]. This model is widely used when formulating objectives and managing trade‐offs. The steps of MOORA are as follows:

I. Describing criteria and alternatives:
II. Framing the decision matrix (*f* *d*_*m*_):
 The decision matrix includes favourable options and defining factors, with positive and negative criteria marked as “*b*_*f*_” and “*n* *b*_*f*_” respectively.
(28) fdm=Nciji=1212,,…c;j=,,……,n,
 
*m* = number of criteria
 
*n* = number of attributes
 
*c* *i* *j* = performance of alternative i under criteria j
III. Normalize criteria:
 Each decisive attribute is normalized using the formula:
(29) Ncij=Cij∑j=1cCij2 Eq……i,
 where *N*_*c* *i* *j*_ = normalized decisive attribute and *C*_*i* *j*_ = decisive attribute.
IV. Weighted score calculation: weighted score of the criteria is calculated using the following formula:
(30) WNCij =Wi ×Ncij.
 
*W* *N*_*C* *i* *j* _ = weighted normalized criteria
V. Distance calculation:
(31) Positive distance Pdj=maxWNCij1×WNCij2,…,…,WNCijn−WNCij,
(32) Negative distance Ndj=WNCij−minWNCij1×WNCij2,…,…,WNCijn.
VI. Normalization of distances:
(33) NPdj=PdjMaxPdj,
(34) NNdj=NdjMaxNdj.
VII. MOORA score calculation:
(35) Ms=∑jmPdj−Ndj.
VIII. Rank alternatives based on MOORA scores:
 In MOORA model, both positive and negative aspects of all alternatives are considered while decision making is for choosing the alternatives.

##### 3.2.2.6. Evaluation Based on Distance From Average Solution (EDAS)

This method was introduced by Keshavarz Ghorabaee et al. in [51]. In the EDAS model, alternatives are ranked by assessing their magnitude of distance from the average solution considering all the alternatives. In this method, both beneficial and nonbeneficial criteria are considered while choosing the alternatives. The greater distance from the average solution means least choice.

I. Describing criteria and alternatives:
II. Framing the decision matrix (FDM):
 The decision matrix comprises preferred alternatives and decisive attributes, with beneficial and nonbeneficial criteria denoted by “*b*_*f*_” and “*n* *b*_*f*_,” respectively.
(36) fdm=Nciji=1212,,…c;j=,,……,n,
 
*m* = number of criteria
 
*n* = number of attributes
 
*c* *i* *j* = performance of alternative i under criteria *j*
III. Normalization of criteria:
 Normalize each criterion value for all alternatives to ensure comparability
 Each decisive criteria are normalized using the formula:
(37) Ncij=Cij∑j=1cCij2.
IV. Calculation of average solution $\left(\overset{¯}{N_{cij}}\right)$:
(38) The average solution Ncij¯=1n∑1nNcij.
V. Compute distance from average solution (CDAS):
 For each alternative i and criterion *j*, calculate the squared distance
(39) CDASNcij=Ncij−Ncij¯2.
VI. Assign weights:
 Assign weights *w*_*c* *i* *j*_ to each criterion based on their importance. Ensure the weights sum up to 1.
VII. Calculation of overall distance from average solution:
(40) CDASi =∑j=1mWcij×CDAScij2.
VIII. Rank alternatives:
 In this model, nearest distance of the alternative from the average solution is considered as the best choice.

##### 3.2.2.7. Selection of Morphometric Factors Influencing Groundwater Scarcity Assessment

The morphometric variables considered for assessing the groundwater scarcity in the Kangsabati River Basin are relevant for groundwater scarcity modelling. The morphometric variable is categorised in beneficial and nonbeneficial factors according to their influences on groundwater storage. The selected variables and their description are shown in Table 1.

**TABLE 1:** Selected morphometric variables for assessing groundwater scarcity.

Key morphometric variables for MADM in groundwater scarcity modelling	The effect of morphometric variables on groundwater availability in a drainage basin	Beneficial and nonbeneficial factors
1. Basin area (A)	Larger basin areas have more opportunities for groundwater recharge due to higher precipitation inputs, reduced surface runoff, greater infiltration capacity and diverse topographic and land‐use characteristics [52, 53].	NB
2. Basin perimeter (P)	A longer perimeter increases the chances of surface water seeping into the ground, thus facilitating groundwater recharge [54].	B
3. Weighted mean bifurcation ratio (*R*_bwm_)	A higher weighted mean bifurcation ratio value indicates a more complex network with fewer smaller tributaries than larger main channels. This complexity influences the distribution of surface runoff and infiltration, impacting groundwater recharge [55].	B
4. Basin length (*L*_*b*_)	Longer basin lengths usually result in longer flow paths for surface water, allowing more time for the water to infiltrate the ground and recharge groundwater supplies [56].	NB
5. Stream order (*S*_*µ*_)	A higher stream order and stream number suggest a greater amount of surface runoff occurring [57].	NB
6. Stream number (*N*_*µ*_)	A higher number of streams means that surface water has more chances to mix with the underground environment. Streams work as channels for water flow, and a more compact network enhances the possibility of water reaching the groundwater system through direct infiltration or bank storage processes [58].	B
7. Stream length (*L*_*µ*_)	Lengthening the stream generally results in delayed peak discharge and prolonged flow duration, allowing water to infiltrate the soil and recharge groundwater aquifers [59].	B
8. Mean stream length $\left({\overset{¯}{L}}_{µ}\right)$	Long stream lengths can intersect with deeper aquifers or geological formations, providing pathways for groundwater recharge through direct infiltration or lateral flow along stream channels [60].	NB
9. Basin width (*W*_*b*_)	Wider basins may have diverse topography, such as floodplains and alluvial deposits. This can increase groundwater recharge by improving infiltration and storage capacity [61].	B
10. Length area relation (*L*_*a* *r*_)	While larger basins with longer stream channels generally provide more extensive areas for possible recharge, the rate of increase in stream length diminishes with basin size, indicating that the additional length may not proportionally improve recharge potential [62].	NB
11. Basin form factor (f)	Basin shape with lower form factor values is more elongated, improving groundwater storage [54].	B
12. Basin circulatory ratio (*R*_*c*_)	A more circular basin shape allows for more efficient groundwater storage [63].	NB
13. Compactness coefficient (*C*_*c*_)	Lower values of compactness coefficient indicate a basin with a more elongated shape, which may facilitate groundwater storage [64].	B
14. Basin elongation ratio (*R*_*e*_)	Changing the shape of the basin to be more elongated could improve groundwater storage [65].	NB
15. Drainage density (*D*_*d*_)	Lower runoff values may indicate more water seeping into the ground through infiltration [66, 67].	B
16. Stream frequency (*F*_*s*_)	Reduced stream flow may indicate a lower surface runoff and a possibility of higher infiltration [68].	B
17. Infiltration number (If)	Higher infiltration rates can improve groundwater recharge by facilitating better groundwater absorption [68].	NB
18. Length of overland flow (*L*_*g*_)	Increasing the length of overland flow paths could improve the process of infiltration and recharge of groundwater [69].	NB
19. Constant channel maintenance (CCM)	Less water is needed to maintain streams, potentially leaving more for groundwater [70].	B
20. Total basin relief (H)	Higher total relief and gradient ratio suggest recharge at higher elevations [71].	B
21. Relative relief (*R*_*R*_)	Regarding groundwater recharge, relative relief may offer valuable insights into potential recharge rates [72].	B
22. Ruggedness number (*R*_*n*_)	A high ruggedness indicates rough terrain, resulting in rapid runoff and less infiltration [73].	B
23. Melton ruggedness number (MRn) (*M* *R*_*n*_)	A high Melton ruggedness number indicates a rugged terrain, resulting in rapid runoff and less infiltration [74].	B
24. Gradient ratio	Gentle slopes can help infiltrate water into the soil, which, in turn, can aid in groundwater storage [75].	B

*Note:* NB: the nonbeneficial factors for groundwater scarcity assessment in drainage basins are considered the factors that favour groundwater recharge. B: the factors that disfavour groundwater recharge are considered beneficial factors for groundwater scarcity assessment in a river basin.

## 4. Results and Discussion

### 4.1. Morphometric Characteristics of the Basin

The derived results of morphometric analysis of the basin give an intensive insight about the hydrogeomorphic characteristics of it. A comprehensive idea about the groundwater status is revealed from the analytical examination of morphometric variables (Table 2).

**TABLE 2:** Morphometric analyses of Kangsabati River Basin.

Sl. No.	Parameters	Formulae	References	Results
*(A) Linear aspects of channel system on drainage network*				
1	Stream of order (*μ*)	(*μ*)	Strahler, 1964	1–7
2	Number of streams (*N μ*)	*N μ*	Horton, R.E., 1945	4143
3	Bifurcation ratio (*R b*)	*R b* = *N μ/*N *μ*+	Schumm, 1956	2.00–6.40
4	Mean bifurcation ratio (*R*_bm_)	bm = *N μ*/N *μ* ῑμ = *L μ/N μ*	Schumm, 1956	3.55
5	Weighted mean bifurcation ratio (*W*_Rbwm_)	*R*_bwm_	Schumm, 1957	4.47
6	Stream length (*L* ῑμ = *L μ/N μ μ* /) in km	ῑμ = *L μ/N μ*	Horton, 1945	6008.96
7	Mean stream length	ῑμ = *L μ/N μ*	Strahler, 1964	36.01
8	Stream length ratio	RL = ῑμ/ῑμ − 1	Horton, 1945	1.86–3.50
9	Mean stream length ratio	ῑμ = *L μ/N μ*	Strahler, 1964	2.01
10	Weighted mean stream length ratio	RLw = ∑Ni∑ (RLi × Ni)	Strahler, 1964	2.27
11	Main channel length (*C*_*l*_) in km	(*C*_*l*_)	Horton, 1945	273.00
12	Channel index (CI)	CI = CL/AL	Muller, 1968	1.45
13	Rho coefficient (q)	*ρ* = *D*_*d*_/*R*_*L*_	Horton, 1945	8.69

*(B) Areal aspects of drainage basin: basin geometry*				
14	Basin area (A) Km^2^	*A μ*	Schumm, 1956	5620.61
15	Basin length (Lb) in km	*L μ*	Schumm, 1956	188.56
16	Basin width (Wb)	Br *μ*	Schumm, 1956	29.81
17	Basin perimeter (P)	P *μ*	Schumm, 1956	787.98
18	Relative perimeter (Pr)	Pr = *A*/P	Schumm, 1956	7.13
19	Length area relation (Lar)	Lar = 1.4 ∗ A0.6	Hack, 1957	248.88
20	Lemniscate’s (k)	*F* = *A*/Lb 2	Chorley et al., 1957	6.33
21	Form factor (F)	*F* = $L_{b}^{2}$ *A A*/$L_{b}^{2}$	Horton, 1945	0.158
22	Circulatory ratio (Rc)	R *c* = 4 Π A/*P* 2	Strahler, 1964	0.114
23	Wandering ratio (Wr)	Wr = CL/Lb	Smart and Surkar, 1967	1.448
24	Fitness ratio (Fr)	Fr = CL/*P*	Melton, 1957	0.346
25	Compactness coefficient (Cc)	Cc = 0.2841 ∗ *P*/A0.5	Gravelius, 1914a, 1914b	2.986
26	Basin elongation ratio (Re)	*R e* = (2 √*A*/Π)/Lb	Schumm, 1956	0.253
27	Drainage density (Dd)	D *μ* = (Σ *L μ/*ΣAμ)	Horton, 1945	1.069
28	Stream frequency (Fs)	F *μ* = (Σ*N μ*/Σ Aμ)	Horton, 1945	0.737
29	Drainage intensity (Di)	Di = Dd/Fs	Faniran, 1968	0.689
30	Infiltration number (If)	If = Fs ∗ Dd	Faniran, 1968	0.788
31	Length of overland flow (Lg)	Lg = 1/Dd ∗ 2	Horton, 1945	0.468
32	Constant channel maintenance (CCM) (sq kms/km)	*C* = 1/*D μ*	Schumm, 1956	0.935
33	Texture ratio (Rt)	*T μ* = *N μ*/*P μ*	Horton, 1945	5.26

*(C) Relief aspects of drainage basin surface*				
34	Height of highest point on watershed (Z)	DEM		659
35	Height on basin mouth (z)	DEM		27
36	Total basin relief (H)	*H* = Z‐z	Strahler, 1952	632
37	Relief ratio (*R*_*h*_)	*R*_*h*_ = *H*/*L*_*b*_	Schumm, 1956	3.35
38	Ruggedness number (*R*_*n*_)	*R*_*n*_ = *D*_*d*_ × *H*	Patton and Baker, 1976	0.676
39	Total contour length (Lc) km	GIS software	—	16397
40	Contour interval (Ic), m	GIS software	—	20
41	Mean slope of the basin (Sm), %	Sm = (Lc X Ic)/A	Chorley, 1969	58.35
42	Average slope	tanØ¯ = NxI/Wk	Wentworth, 1930	Total basin’s average slope zone‐wise
43	Slope in degree	GIS software		0–57.89
44	Melton ruggedness number (MR_n_)	MRn = *H*/√*A*	Melton, 1957	8.43
45	Gradient ratio	Rg = H/*L*_*b*_	Sreedevi, 2005	3.35
46	Relative relief (RR)	RR = *H*_max_ − *H*_min_	Smith, 1935	Total basin’s relative relief zone‐wise
47	Dissection index (DI)	DI = *H*_*m*_/*H*_*r*_	Dov Nir, 1957	Total basin’s dissection index relief zone‐wise

#### 4.1.1. Linear Aspects of Channel System on Drainage Network

A complex drainage network has evolved in the Kangsabati River Basin. There are 4143 streams in the basin. Applying Strahler’s scheme of stream ordering reveals that the streams range from 1^st^ order to 7^th^ order. The hierarchical nature of stream networks exposes that lower‐order streams contribute more water to the main channel. The branching of the channels extracted by the bifurcation ratio (Rb) ranges from 2.00 to 6.40. Thus, channel branching in this basin is moderate to high. A balanced branching pattern in the drainage network is indicated by the weighted mean bifurcation ratio (Rbwm), which has a derived value of 4.47, and the mean bifurcation ratio, which has a value of 4.14. The basin summation of stream length (Lµ) is measured as 6088.96 km, while the mean stream length is 36.01 km. The extracted stream length ratio (RL) is 2.34, establishing the law of stream length, i.e., the length of the stream increases with increasing the order of the stream. The overall distribution of stream length in the basin is indicated by the mean stream length ratio, which is calculated as 2.77. The Rho value of the basin is 8.69, which exposes that the basin has a high degree of drainage network development and stream distribution. The extracted results of linear aspects of the basin denote a complex drainage network. This finding gives a better understanding of the hydrogeomorphic status of the basin.

#### 4.1.2. Areal Aspects of Drainage Basin: Basin Geometry

Assessing hydrological behaviours in a drainage basin requires a better understanding of the basin’s geometric and morphometric characteristics. The extracted results of the areal aspects of the Kangsabati River Basin give significant insight into its hydrogeomorphic characteristics. The total area of Kangsabati River Basin is about 5620.61 km^2^; such a large basin indicates its vast catchment area has significant water resource potential. It also denotes that the basin is at high flood risk while extreme rainfall occurs. The length of the basin (*L*_*b*_) is measured as 188.56 km; such a relatively long basin tends to take a long time for peak discharge, which would take a comparatively long lag period to cause flood events in the lower segment of the basin. The width of the basin (*W*_*b*_) is 29.81 km, which is comparatively lesser than the basin length, indicating more elongation. The perimeter of the basin is 787.98 km, indicating the irregularities in the basin shape and the tendency of more runoff in it. The irregularity in basin boundary and heterogeneity in the terrain are indicated by the high value of the relative perimeter (*P*_*r*_) of the basin, which is measured at about 7.13. The basin’s length‐area ratio (*L*_ar_) value is 248.88, which signifies a long time for the runoff concentration and not the sudden potentiality of flood in the basin. The high lemniscate (k) value measured as 6.33 indicates a longer runoff time and lesser flash flood risk. The derived value of form factor (F) is 0.158, indicating the elongated basin shape characterized by low peak flow but long duration flood events in the basin. The circularity ratio (*R*_*c*_) of the basin is 0.114, which experiences the basin with dispersed peak flow over time. The moderate meandering nature of the channel in the basin is indicated by the wandering ratio (*W*_*r*_), measured as 1.448. Thus, the decreased water velocity leads to sediment deposition in the lower segment of the basin. The extracted fitness ratio (*F*_*r*_) of the basin is 0.346, which exposes the restrictive stream development due to structural control or smooth topography of the basin. The irregularities in basin shape and high chances of water logging conditions are due to low drainage efficiency, as the compactness coefficient (*C*_*c*_) is only 2.986. The elongation ratio of the basin is 0.253, signifying the elongated nature of the basin and exposing the long‐lasting nature of the flood. The moderate potentiality of infiltration in the Kangsabati River Basin is indicated by the moderate drainage density (*D*_*d*_) measured as 1.069 km/km^2^. The low stream frequency (*F*_*s*_) of the basin indicates comparatively low surface runoff due to this presence. The basin’s drainage intensity (*D*_*i*_) is 0.689, which denotes the balanced runoff and infiltration. The calculated infiltration number (If) is 0.788, indicating the low potentiality of infiltration in the basin. The length of overland flow (*I*_*g*_) of the basin is 0.468 km, which signifies that the water from the tributaries does not take a longer time to reach the main channel. The value of the basin’s constant channel maintenance (CCM) is 0.935 km/km^2^, indicating the requirement of less area of each channel segment for the development of efficient drainage development. The closeness of streams and well‐developed drainage in the basin are indicated by the texture ratio (*R*_*t*_), which is measured as 5.26.

#### 4.1.3. Relief Aspects of Drainage Basin

The expression of topographic details has been revealed through the intensive investigation of the relief aspects of the Kangsabati River Basin. At the source of Kangsabati River, the height elevation (Z) is extracted as 659 m, while at the mouth of the river, the lowest elevation (z) is found as 27 m (Figure 3a). Thus, the basin’s total relief (H) is 632 m. Such a high relief value reveals the elevation variation within the basin, leading to high erosional activities, especially in the upper segment of the basin. The extracted relief ratio (*R*_*h*_) is 3.35, indicating the high to moderate steepness of the basin. Moderately rugged terrain conditions are exposed to the derived ruggedness number (Rn), which has a value of 0.676. The basin’s mean slope (*S*_*m*_) is 58.35%, indicating the steepness of the relief, which enhances runoff and reduces groundwater recharge. The slope of the basin varies from almost 0° to 57° in the lower and upper sections of the basin, respectively (Figure 3c). Such slope conditions indicate a high rate of vertical erosion in the upstream and depositional activities in the lower segment of the basin. The extracted Melton ruggedness number (MR_n_) of the basin is 8.43, which signifies that the basin is highly susceptible to intensive runoff when heavy rainfall occurs. The derived gradient ratio (3.35) of the basin indicates a high potentiality of erosion along the channels. The calculated dissection index indicates the highly dissected landscape in the basin, which is 0.95. The relative relief (RR) of the basin ranges from 0 to 365 m, while the dissection index (DI) varies between 0 and 0.42, indicating low to moderate terrain dissection (Figure 4). These morphometric characteristics play an important role in controlling hydrological processes and, consequently, water stress conditions within the basin.

**FIGURE 3:**
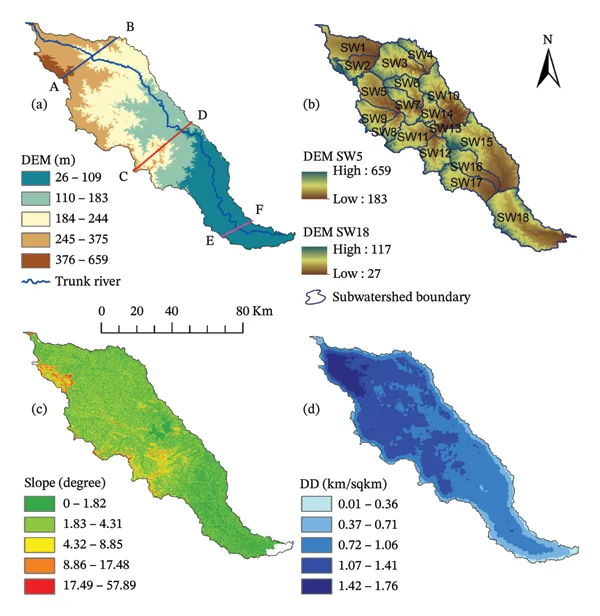
Relief aspects of Kangsabati River Basin. (a) DEM with Trunk River and cross sections, (b) subwatershed boundary, (c) slope and (d) drainage density.

**FIGURE 4:**
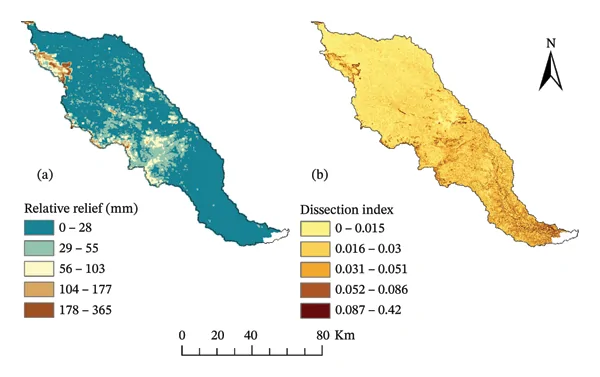
(a) Relative relief and (b) dissection index.

#### 4.1.4. Hydrogeomorphological Assessment Based on Cross‐Sectional Analysis

To excavate the hydrogeological characteristics of Kangsabati River through cross‐section analysis, three cross sections have been selected, namely, AB, CD and EF, at the upstream, middle course and lower course, respectively (Figure 5). The detailed analysis of the cross sections provides remarkable insights into the Kangsabati River’s longitudinal evolution, erosional dynamics and depositional characteristics. The cross‐section A‐B at upstream or headwater zone reveals steep slopes with notable variation in elevation which ranges from 600 to 250 m downstream. The sharp breaks of slope, deep cuts and irregularities in relief are observed in the profile depicting the evidence of intense vertical erosion. The rugged terrain has been carved by the processes of hydraulic action and abrasion. A youthful stage of fluvial development is indicated by the steep gradient along with high flow velocity. Structural control is found on channel development which is confined within hard rock formation. In the segment, fluvial deposits are absent. The river’s middle reach cross‐section C‐D displays gently undulating profile with elevation ranges from 300 to 100 m. This section shows the evidences of transition from vertical to lateral erosion. The presence of intermontane depression and terrace remnants in the profile, characterized by a meandering channel, reveals a state of equilibrium between erosional and depositional processes. The evidences of river’s increasing load and adjustment to lithological variations and base‐level changes are observed from the development of alluvial fans and piedmont plains along this section.

**FIGURE 5:**
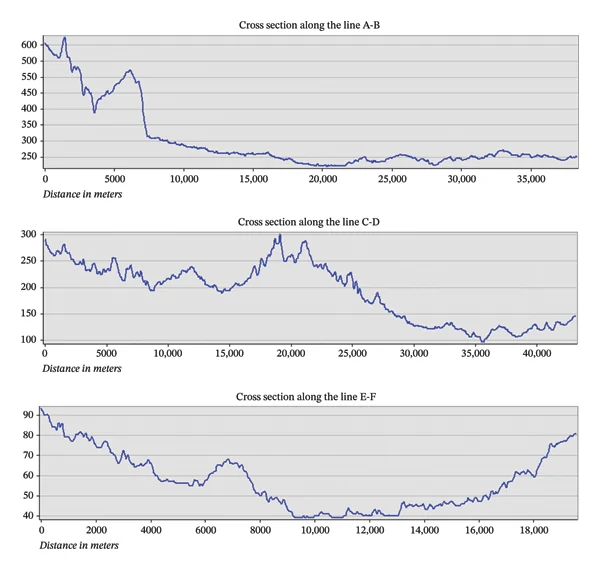
Kangsabati River’s cross sections AB, CD and EF at the upstream, middle course and lower course, respectively.

The E‐F cross section on the lower course of the Kangsabati River is characterised by gentle slope gradient with elevation ranging from 40 to 90 m. A broad floodplain morphology is reflected by the concave upward profile in this cross section. Existence lateral accretion, meandering channels, natural levees and flood basins along this section reveal the dominance of depositional processes. The river’s old stage is indicated by the low relief and extensive alluvial cover along this section. Evidences of periodic inundation and floodplain evolution through repeated overbank depositions indicated by the formation of flat valleys along this cross‐section. From the comparative analysis of the cross‐sections, it is inferred that there is a dynamic equilibrium in fluvial processes evolved by topography, lithology and hydrological regime of Kangsabati River. The river’s geomorphic maturity, sedimentological adjustment and gradual evolution toward a graded profile lead to a stabilized fluvial system.

### 4.2. Modelling Groundwater Scarcity Prioritization in the Kangsabati River Basin

In this study, groundwater paucity status in the Kangsabati River Basin has been estimated based on hydrological characteristics derived through the analysis of the morphometric variables of the basin (Table 3, Figure 6). The prioritization of subwatersheds based on the degree scarcity of groundwater in the basin has been decided through the implication of six methods of MCDA, viz, TOPSIS, VIKOR, SAW, CoCoSo, MOORA and EDAS. This helps in understanding the prioritization of subwatersheds in the basin in a more rational way.

**TABLE 3:** Subwatershed‐wise selected morphometric variables for groundwater stress zonation in the Kangsabati River Basin.

Parameters	1SW	2SW	3SW	4SW	5SW	6SW	7SW	8SW	9SW	10SW	11SW	12SW	13SW	14SW	15SW	16SW	17SW	18SW
Area	385.7	197.8	416.9	168.4	306.3	192.3	206.7	115.6	248.4	413.7	293.1	263.8	51.9	205.0	782.3	256.6	330.2	785.8
Perimeter	196.1	104.5	155.4	109.1	133.9	120.5	118.2	83.9	121.5	171.8	127.6	115.5	57.0	116.7	252.7	153.4	159.0	246.3
Rbwm	4.4	4.5	4.6	5.2	4.2	4.7	5.1	5.2	4.4	4.2	4.9	4.3	4.0	4.5	4.4	4.9	3.8	4.3
Lb in km	40.2	21.7	30.3	24.4	29.1	26.4	23.8	18.4	22.7	39.5	28.9	22.7	11.6	26.7	38.7	35.8	33.3	52.5
Stream order	5.0	5.0	6.0	4.0	5.0	4.0	6.0	4.0	5.0	5.0	4.0	5.0	5.0	6.0	7.0	4.0	5.0	5.0
Stream number (Nu)	468.0	262.0	384.0	151.0	334.0	158.0	161.0	119.0	251.0	285.0	259.0	237.0	37.0	140.0	19.0	151.0	201.0	200.0
Stream length (Lu), km	565.4	340.8	603.9	187.7	462.7	216.0	302.9	140.8	345.6	487.4	341.1	315.5	108.7	280.8	990.1	246.5	336.2	695.9
Mean stream length of order	113.1	68.2	100.7	46.9	92.6	54.0	50.5	35.2	69.1	97.5	85.3	63.1	21.7	46.8	141.5	61.6	67.3	139.2
Width of basin of order (μ)	9.6	9.1	13.8	6.9	10.5	7.3	8.7	6.3	11.0	10.5	10.1	11.6	4.5	7.7	20.2	7.2	9.9	15.0
Length area relation (Lar)	49.9	33.4	52.3	30.3	43.4	32.9	34.3	24.2	38.3	52.0	42.3	39.7	15.0	34.1	76.2	39.1	45.4	76.4
Basin form factor	0.2	0.4	0.5	0.3	0.4	0.3	0.4	0.3	0.5	0.3	0.4	0.5	0.4	0.3	0.5	0.2	0.3	0.3
Basin circulatory ratio	0.1	0.2	0.2	0.2	0.2	0.2	0.2	0.2	0.2	0.2	0.2	0.3	0.2	0.2	0.2	0.1	0.2	0.2
Compactness coefficient (Cc)	2.8	2.1	2.2	2.4	2.2	2.5	2.3	2.2	2.2	2.4	2.1	2.0	2.3	2.3	2.6	2.7	2.5	2.5
Basin elongation ratio	0.3	0.4	0.4	0.3	0.4	0.3	0.4	0.4	0.4	0.3	0.4	0.5	0.4	0.3	0.5	0.3	0.4	0.3
Drainage density (Dd)	1.5	1.7	1.5	1.1	1.5	1.1	1.5	1.2	1.4	1.2	1.2	1.2	2.1	1.4	1.3	1.0	1.0	0.9
Stream frequency (Fs)	1.2	1.3	0.9	0.9	1.1	0.8	0.8	1.0	1.0	0.7	0.9	0.9	0.7	0.7	0.0	0.6	0.6	0.3
Infiltration number (If)	1.8	2.3	1.3	1.0	1.7	0.9	1.1	1.3	1.4	0.8	1.0	1.1	1.5	0.9	0.0	0.6	0.6	0.2
Length of overland flow (Lg)	0.3	0.3	0.4	0.5	0.3	0.5	0.3	0.4	0.4	0.4	0.4	0.4	0.2	0.4	0.4	0.5	0.5	0.6
Constant channel maintenance (sq Kms/km)	0.7	0.6	0.7	0.9	0.7	0.9	0.7	0.8	0.7	0.9	0.9	0.8	0.5	0.7	0.8	1.0	1.0	1.1
Total basin relief	405.0	402.0	128.0	98.0	476.0	131.0	201.0	196.0	374.0	134.0	301.0	292.0	105.0	148.0	251.0	247.0	246.0	90.0
Relative Relief	15.6	28.8	10.2	11.4	22.6	11.0	14.0	21.4	25.2	6.7	15.2	19.0	20.3	10.5	7.9	8.5	9.1	2.2
Ruggedness number	0.6	0.7	0.2	0.1	0.7	0.2	0.3	0.2	0.5	0.2	0.4	0.4	0.2	0.2	0.3	0.2	0.3	0.1
Melton ruggedness number (MR_n_)	20.6	28.6	6.3	7.6	27.2	9.5	14.0	18.2	23.7	6.6	17.6	18.0	14.6	10.3	9.0	15.4	13.5	3.2
Gradient ratio	10.1	18.5	4.2	4.0	16.3	5.0	8.5	10.7	16.5	3.4	10.4	12.9	9.1	5.6	6.5	6.9	7.4	1.7

**FIGURE 6:**
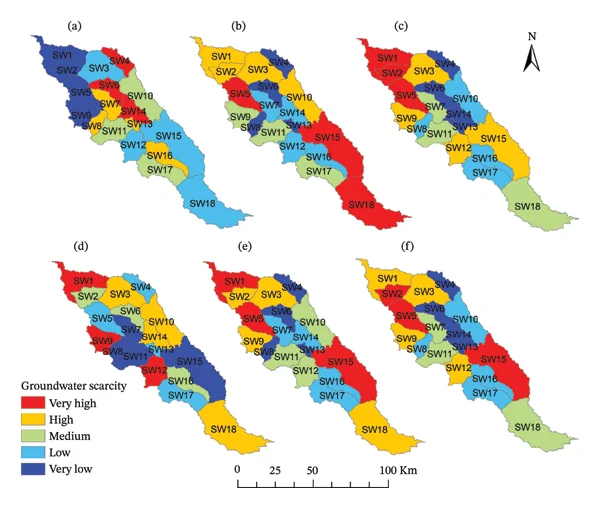
Groundwater scarcity prioritization in the Kangsabati River Basin: (a) TOPSIS, (b) VIKOR, (c) SAW, (d) CoCoSo, (e) MOORA and (f) EDAS.

#### 4.2.1. TOPSIS Model: Analysing Groundwater Scarcity in the Basin

The subwater shades (SW) of the Kangsabati River Basin have been ranked based on the severity of scarcity of groundwater while decision has been made through the TOPSIS model of MCDA. In the TOPSIS model, the alternatives are evaluated based on closeness near the PIS and remoteness from the NIS. In this method, the value of the relative closeness coefficient is considered to determine the alternative for prioritizing the choice. In this study, the subwatersheds are prioritized according to the severity of groundwater scarcity. Thus, in this analysis, the subwatershed with the high value of relative closeness coefficient indicates more groundwater scarcity and vice versa. The analysis of the TOPSIS model in this study reveals that the SW4 suffers the highest groundwater scarcity as its value of the relative closeness coefficient is highest, i.e., 0.728 (Figure 6a). On the other hand, the lowest value of the relative closeness coefficient is 0.387, which is found in the case of SW5 (Table 4).

**TABLE 4:** Comparative analysis of different models of MCDA for groundwater scarcity assessment.

Subwatershed	TOPSIS model		VIKOR		SAW		CoCoSo		MOORA		EDAS	
	Value	Ranking	Value	Ranking	Value	Ranking	Value	Ranking	Value	Ranking	Value	Ranking
SW1	0.386	18	0.645	4	0.705	3	0.073	2	0.087	1	0.131	4
SW2	0.419	16	0.540	7	0.714	2	0.072	3	0.065	6	0.146	2
SW3	0.526	13	0.550	6	0.623	7	0.062	6	0.067	5	0.064	6
SW4	0.728	1	0.253	17	0.469	18	0.042	16	0.036	17	−0.133	17
SW5	0.387	17	0.672	3	0.719	1	0.077	1	0.077	3	0.144	3
SW6	0.717	2	0.294	15	0.479	16	0.042	17	0.039	15	−0.131	16
SW7	0.640	7	0.335	13	0.551	10	0.051	10	0.045	12	−0.018	10
SW8	0.644	6	0.279	16	0.525	13	0.050	12	0.038	16	−0.067	13
SW9	0.462	15	0.533	8	0.674	4	0.067	4	0.064	7	0.111	5
SW10	0.605	9	0.550	5	0.547	11	0.051	11	0.059	8	−0.052	11
SW11	0.549	10	0.484	10	0.605	8	0.059	8	0.058	9	0.025	8
SW12	0.538	12	0.453	11	0.624	6	0.060	7	0.055	10	0.055	7
SW13	0.688	4	0.151	18	0.469	17	0.065	5	0.023	18	−0.143	18
SW14	0.709	3	0.312	14	0.496	15	0.046	14	0.040	14	−0.102	15
SW15	0.482	14	0.998	1	0.669	5	0.048	13	0.082	2	0.149	1
SW16	0.674	5	0.413	12	0.506	14	0.055	9	0.044	13	−0.094	14
SW17	0.631	8	0.492	9	0.542	12	0.036	18	0.051	11	−0.062	12
SW18	0.540	11	0.943	2	0.586	9	0.043	15	0.068	4	0.009	9

#### 4.2.2. VIKOR Model: Analysing Groundwater Scarcity in the Basin

The VIKOR is one of the models of MCDA where a compromise solution is needed to choose from the conflicting criteria or objectives. In this model, the choices are ranked according to the CEI value. The highest CEI value means the best choice among the options and vice versa. The VIKOR model has been applied to prioritize the subwatersheds according to the harsh groundwater scarcity within the Kangsabati River Basin. From the extracted results of the VIKOR model of this study, it is found that the highest CEI value is 0.998, which is in the SW15. Thus, SW15 has the highest groundwater scarcity among the subwatersheds within the Kangsabati River Basin. On the other hand, SW13 has the lowest severity of groundwater scarcity as it has the lowest CEI value, i.e., 0.151 (Figure 6b). Thus, SW15 attracts top priority for taking immediate management of groundwater resources. However, in all subwatersheds, management and restoration of groundwater are necessary.

#### 4.2.3. SAW Model: Analysing Groundwater Scarcity in the Basin

SAW is a widely used model of the MCDA. This model is convenient for decision making when multicriteria are involved in the alternatives. In this model, a predetermined weight is assigned to each criterion multiplied by each respective criterion, and the results are derived by summing them. The highest value of SAW against the alternative among the given alternatives is considered the best choice and vice versa. Ranking of the subwatersheds in Kangsabati River Basin according to the sternness of groundwater scarcity has been done by applying the SAW model of MCDA. The calculated results of the SAW model expose that the highest weighted score is 0.719 against the SW5, which denotes the highest prioritization in terms of groundwater scarcity among all the subwatersheds within the Kangsabati River Basin (Figure 6c). The scarcity of groundwater is insignificant in SW4 as its weighted score is 0.469, the lowest value among all watersheds within the basin (Table 4). The analysis revealed that groundwater management is essential in the Kangsabati River Basin.

#### 4.2.4. CoCoSo Model: Analysing Groundwater Scarcity in the Basin

The CoCoSo model is found to be an effective use when complex scenario is raised in MCDA. This is a structured model in which multiple criteria are balanced to reach a compromise solution. In this model, the higher score indicates the better alternatives. The subwatershed within the Kangsabati River Basin has been prioritized based on the degree of scarcity of groundwater. The results of the CoCoSo model in this study depict that SW5 achieved the highest score and signified the top scarcity of groundwater (Figure 6d). On the other hand, SW4 receives the lowest score, i.e., 0.46856, denoting minimal groundwater scarcity among all the subwatersheds within the Kangsabati River Basin (Table 4). However, groundwater management is necessary for all areas within the basin to overcome the problem of groundwater scarcity.

#### 4.2.5. MOORA: Analysing Groundwater Scarcity in the Basin

The MOORA model of the MCDA is widely used in engineering, business and operational research to decide alternatives based on multiple conflicting criteria. In this systematic framework model, both beneficial and nonbeneficial criteria are taken into consideration. In this model, overall performance scores by the alternatives are judged. The highest positive score is considered the best alternative. The model has been applied to assess the groundwater scarcity in the subwatersheds of Kangsabati River, and subwatersheds were ranked according to MOORA score in descending order (Table 4). The derived results depict that SW1 has the highest scarcity (3134.27) of groundwater among all watersheds. On the other hand, SW13 has the least severity of groundwater scarcity (Figure 6e).

#### 4.2.6. Evaluation Based on Distance From Average Solution (EDAS): Analysing Groundwater Scarcity in the Basin

The EDAS model is a systematic way for ranking the alternatives according to their positive and negative distance from an average reference point. This method suits MCDA when several criteria are involved in making the best choice. Applying this model to assessing the groundwater scarcity status among the subwatershed in the Kangsabati River basis, it is revealed that SW15 scored the lowest (0.148) and drew the highest priority regarding groundwater scarcity (Figure 6f). The highest score achieved by SW13 signifies the lowest priority regarding groundwater scarcity (Table 4).

#### 4.2.7. Comparative Analysis of Multicriteria Decision Analysis Techniques for Groundwater Scarcity Assessment in Kangsabati River Basin

A comparative study among the derived results regarding the prioritization of subwatersheds within the Kangsabati River Basin according to the degree of groundwater scarcity of selected MCDA models showed their uniqueness owing to their methodological differences. A similarity was observed in the derived results between TOPSIS and VIKOR models for prioritizing subwatersheds within the Kangsabati River Basin according to the degree of scarcity of groundwater, as both models depict that SW4 and SW1 have the highest and lowest priority, respectively. As per the SAW model, SW5 and SW achieved the highest and lowest priority regarding the severity of groundwater scarcity. Meanwhile, the CoCoSo model indicates the highest priority for SW5 and the weakest for SW1. On the other hand, the MOORA model depicts the highest priority to SW1. While the EDAS model depicts the highest priority as SW1 (Table 4), a critical analysis of the extracted results of different MCDA methods reflects that most models denote that SW4 steadily highest and SW1 lowest prioritization in terms of severity of groundwater scarcity. The diversified ranking of subwatersheds by the different models of MCDA suggests the need for multiple integrated methods of MCDA to assess the groundwater status in the Kangsabati River Basin.

The comparative MADM evaluation reveals a strong spatial consensus across diverse algorithmic architectures, pointing to SW5, SW15, SW1 and SW2 as the absolute core zones of groundwater scarcity within the Kangsabati River Basin. Distance‐from‐reference models like VIKOR and EDAS place SW15 at the apex of vulnerability, while product‐sum matrices such as SAW and CoCoSo identify SW5 as the primary risk zone, showcasing how different normalization workflows interact with identical morphometric data (Table 5). This clustering of high scarcity in upstream subwatersheds perfectly mirrors geomorphic features where steep gradients and high ruggedness numbers accelerate surface runoff at the expense of vertical infiltration. Conversely, the models consistently track SW13, SW4 and SW6 as the lowest scarcity anchors, matching downstream transitions where lower channel gradients naturally support enhanced bank storage and river‐aquifer connectivity (Table 5). By mitigating individual mathematical biases, this parallel multimodel approach confirms that structural conservation measures, such as subsurface managed aquifer recharge (MAR), must be strategically prioritized within SW5, SW15, SW1 and SW2 to ensure long‐term watershed sustainability.

**TABLE 5:** Summary of absolute scarcity bounds and corresponding high‐to‐low priority subwatershed zoning across the utilized MADM architectures.

Model	Highest scarcity	Lowest scarcity	Highest scarcity subwatershed	Lowest scarcity subwatershed
TOPSIS	SW4 (0.728)	SW5 (0.386)	SW4, SW6, SW14	SW1, SW2, SW4, SW9
VIKOR	SW15 (0.998)	SW13 (0.151)	SW5, SW15, SW18	SW4, SW6, SW8
SAW	SW5 (0.719)	SW4 (0.469)	SW1, SW2, SW5	SW4, SW6, SW13, SW14
CoCoSo	SW5 (0.077)	SW17 (0.036)	SW1, SW9, SW12	SW7, SW8, SW11, SW15
MOORA	SW1 (0.087)	SW13 (0.023)	SW1, SW5, SW15	SW4, SW6, SW8, SW13
EDAS	SW15 (0.149)	SW13 (−0.143)	SW2, SW5, SW15	SW4, SW6, SW13, SW14

The severe groundwater scarcity mapped in high‐elevation upstream subwatersheds like SW1 and SW2 perfectly aligns with hard‐rock terrain dynamics studied in the Indian Deccan Traps and regional plateau belts by Choudhari et al. [54], where steep relief ratios combined with low channel maintenance accelerate overland flash‐flow at the cost of subsurface infiltration. Conversely, our discovery of low‐stress environments in downstream zones like SW18 mimics consistent patterns established in the Nile and Blue Nile systems, where thick alluvial fan expansion offers massive lateral bank storage and induced stream‐aquifer infiltration corridors. A notable divergence occurs in our VIKOR/EDAS simulations where SW15 emerges as a high‐stress block; this deviation is driven by localized high drainage density coupled with flat geometry, causing intense surface gathering but low transmission into deeply consolidated horizons, a pattern structurally reminiscent of localized sub‐basin constraints identified by Halder et al. [20].

### 4.3. Longitudinal Profile and SL Index in Relation to Water Stress

The longitudinal profile of the main channel exhibits a pronounced concave‐up geometry and closely follows an exponential decay function, as indicated by a high coefficient of determination (*R* ^2^ = 0.9854) (Figure 7a). Such a profile reflects an overall graded condition of the river system, suggesting long‐term adjustment between channel slope, discharge and sediment load. However, the steep elevation drop observed in the upstream reaches indicates high stream power and rapid energy dissipation, which strongly influences surface‐subsurface hydrological interactions.

**FIGURE 7:**
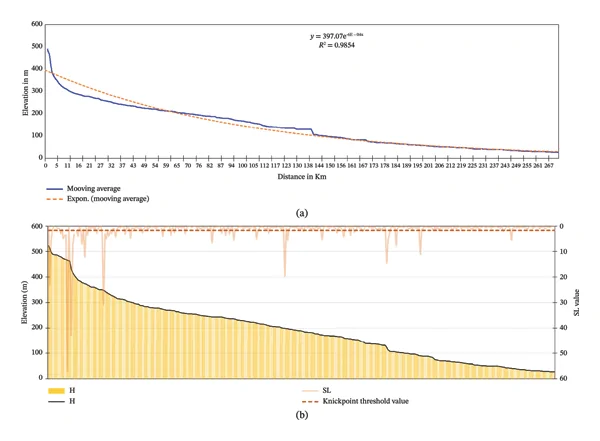
(a) Longitudinal river profile with exponential fit showing overall graded conditions; (b) spatial variation of stream length‐gradient (SL) index along the river profile highlighting knickpoints and zones of elevated groundwater stress.

The spatial distribution of the SL index along the river profile reveals significant local variability superimposed on the basin‐scale graded condition [76]. Several reaches exhibit SL values exceeding the defined knickpoint threshold, indicating abrupt changes in the channel gradient (Figure 7b). These anomalous segments correspond to zones of enhanced channel incision and elevated stream power. From a hydrogeological perspective, such reaches are indicative of reduced groundwater recharge potential, as steep gradients promote rapid surface runoff and limit infiltration opportunities. Additionally, channel down cutting in these segments is likely to induce a lowering of the adjacent groundwater table, thereby weakening river‐aquifer connectivity and reducing base flow contribution.

Knickpoints identified along the longitudinal profile represent geomorphically unstable reaches that may be associated with lithological contrasts, structural controls or tectonic influences. These features can act as hydraulic discontinuities within the basin, facilitating preferential groundwater outflow or vertical leakage, ultimately contributing to localized groundwater stress. The clustering of high SL values in the upstream and mid‐basin reaches suggests that groundwater stress is spatially concentrated rather than uniformly distributed across the basin.

In contrast, downstream reaches are characterized by lower SL values and gentler channel gradients, reflecting reduced stream power and a more stable fluvial regime. These conditions favour enhanced bank storage, induced recharge and sustained groundwater‐surface water interaction, indicating comparatively lower groundwater stress. The progressive downstream decline in SL values therefore highlights a transition from stressed hydrogeological conditions in the upper basin to relatively favourable recharge environments in the lower basin.

So, the integration of longitudinal profile analysis with SL index computation demonstrates that hydromorphometric indicators can effectively identify spatial variations in groundwater stress. Even in the absence of dense groundwater observation data, elevated SL values and knickpoints serve as reliable proxies for zones of enhanced groundwater depletion risk. This approach provides a valuable framework for groundwater stress assessment in data‐scarce river basins and offers important insights for sustainable water resource management and basin‐scale planning.

## 5. Conclusions

Significant findings have been derived from the study of the morphological and hydrological characteristics of the Kangsabati River Basin. The study shows that the basis has an organized drainage network with variable bifurcation ratio and stream length. The stream hierarchy within the basin signifies the hydrological dynamics that are important for managing the basin’s water resources. The analysis of geomorphic parameters provides an important understanding of flood potentials and the availability of water in the basin. Such a better understanding is essential for adopting good strategies for the management of water resources and the conservation of the environment in the basin. For the sustainable development of watershed planning, the hydrogeomorphic features of the basin must be understood through the analysis of relief aspects of the basin.

A limitation of this study is its reliance on static surface morphometrics without real‐time transient deep‐aquifer storage telemetry. Acknowledging this, we recommend that high‐priority hard‐rock segments (SW1, SW2 and SW5) bypass generic check‐dams and transition to subsurface MAR configurations, such as inverted injection shafts that pierce through laterite cap‐rock layers. Furthermore, mid‐basin sectors experiencing channel downcutting must emphasize vegetative bio‐bunding and macrowatershed level contour trenching to systematically lower stream‐gradient velocities and restore declining shallow water tables, mirroring successful conservation models demonstrated in recent hydrogeological research.

The prioritization of the subwatersheds according to the severity of the crisis of groundwater in the basin through the analysis of different methods of MCDA, viz, TOPSIS, VIKOR, SAW, CoCoSo, MOORA and EDAS, gives a rational way to understand the precise scenario and select better alternatives. 8 subwatersheds (SW1, 2, 4, 5, 6, 14, 15 and 18) are highly vulnerable and require strategic prioritization. The inherent complexity of the basin responsible for differential groundwater scarcity in the subwatersheds within the basin is expressed by the different models of MCDA. The study suggests the need to adopt better strategies for managing groundwater resources in the basin that are suited to the environmental condition of the basin’s landscape. An integrated multidisciplinary and holistic approach is to be implemented in the Kangsabati River Basin to restore and manage the basin’s water resources.

## Author Contributions

Sanjay Saha: writing–original draft, conceptualization and methodology. Menuka Gautam: methodology, conceptualization and writing–review and editing. Jayanta Das: methodology, conceptualization, writing–review and editing, supervision and resources.

## Funding

No funding was received for conducting this study.

## Disclosure

All authors reviewed the manuscript.

## Conflicts of Interest

The authors declare no conflicts of interest.

## Data Availability

The data that support the findings of this study are available from the corresponding author upon reasonable request.

## References

[1] Hosseiny S. H. , Bozorg-Haddad O. , and Bocchiola D. , Water, Culture, Civilization, and History, Economical, Political, and Social Issues in Water Resources, 2021, Elsevier, 189–216, 10.1016/B978-0-323-90567-1.00010-3.

[2] Yao J. , Xu N. , Wang M. et al., SWOT Satellite for Global Hydrological Applications: Accuracy Assessment and Insights Into Surface Water Dynamics, International Journal of Digital Earth. (August 2025) 18, no. 1, 10.1080/17538947.2025.247.

[3] Das A. , Applying the Water Quality Indices, Geographical Information System, and Advanced Decision-Making Techniques to Assess the Suitability of Surface Water for Drinking Purposes in Brahmani River Basin (BRB), Odisha, Environmental Science & Pollution Research. (March 2025) 32, no. 23, 13638–13673, 10.1007/s11356-025-36329-z. 40164907

[4] Crow B. and Sultana F. , Gender, Class, and Access to Water: Three Cases in a Poor and Crowded Delta, Society & Natural Resources. (September 2002) 15, no. 8, 709–724, 10.1080/08941920290069308.

[5] Roy P. K. , Basak S. K. , Mohinuddin S. , Roy M. B. , Halder S. , and Ghosh T. , Modelling Groundwater Potential Zone Using Fuzzy Logic and Geospatial Technology of an Deltaic Island, Modeling Earth Systems and Environment. (November 2022) 8, no. 4, 5565–5584, 10.1007/s40808-022-01392-9.

[6] Shah T. , Roy A. D. , Qureshi A. S. , and Wang J. , Sustaining Asia’s Groundwater Boom: An Overview of Issues and Evidence, Natural Resources Forum. (May 2003) 27, no. 2, 130–141, 10.1111/1477-8947.00048.

[7] Cheo A. E. , Ibrahim B. , and Tambo E. G. , Groundwater Resources Development for Livelihoods Enhancement in the Sahel Region, Groundwater for Sustainable Livelihoods and Equitable Growth, 2022, 1st edition, CRC Press, London, 25–61, 10.1201/9781003024101-2.

[8] Mukherji A. and Shah T. , Groundwater Socio-Ecology and Governance: A Review of Institutions and Policies in Selected Countries, Hydrogeology Journal. (March 2005) 13, no. 1, 328–345, 10.1007/s10040-005-0434-9.

[9] Jhariya M. K. , Banerjee A. , and Meena R. S. , Importance of Natural Resources Conservation: Moving Toward the Sustainable World, Natural Resources Conservation and Advances for Sustainability, 2022, Elsevier, 3–27, 10.1016/B978-0-12-822976-7.00027-2.

[10] Machiwal D. , Jha M. K. , and Mal B. C. , Assessment of Groundwater Potential in a Semi-Arid Region of India Using Remote Sensing, GIS and MCDM Techniques, Water Resources Management. (March 2011) 25, no. 5, 1359–1386, 10.1007/s11269-010-9749-y.

[11] Mallick J. , Singh C. K. , Al‐Wadi H. et al., Geospatial and Geostatistical Approach for Groundwater Potential Zone Delineation, Hydrological Processes. (January 2015) 29, no. 3, 395–418, 10.1002/hyp.10153.

[12] Mengistu T. D. , Chung I. M. , Chang S. W. et al., Challenges and Prospects of Advancing Groundwater Research in Ethiopian Aquifers: A Review, Sustainability. (October 2021) 13, no. 20, 10.3390/su132011500.

[13] Lei H. , Meng L. , and Mei Y. , Delineation and Volume Calculation of Groundwater Reserve Based on AHP-EWM: A Case Study of Fuyang City, China, LHB. (December 2025) 111, no. 1, 10.1080/27678490.2025.2565220.

[14] Pradhan A. M. S. , Silwal S. , Kaini S. , and Shrestha S. , Unveiling Groundwater Potential Zones in the Hilly Region of Central Nepal Using Logistic Regression and Random Forest Modeling, Hydrogeology Journal. (August 2025) 33, no. 5, 1465–1482, 10.1007/s10040-025-02932-0.

[15] Matos C. R. , Carneiro J. F. , and Silva P. P. , Overview of Large-Scale Underground Energy Storage Technologies for Integration of Renewable Energies and Criteria for Reservoir Identification, Journal of Energy Storage. (February 2019) 21, 241–258, 10.1016/j.est.2018.11.023.

[16] McWhorter D. B. and Sunada D. K. , Ground-Water Hydrology and Hydraulics, 1977, Water Resources Publications, Fort Collins, Colo.

[17] Sophocleous M. , Interactions Between Groundwater and Surface Water: The State of the Science, Hydrogeology Journal. (February 2002) 10, no. 1, 52–67, 10.1007/s10040-001-0170-8.

[18] Guduru J. U. and Jilo N. B. , Groundwater Potential Zone Assessment Using Integrated Analytical Hierarchy Process-Geospatial Driven in a GIS Environment in Gobele Watershed, Wabe Shebele River Basin, Ethiopia, Journal of Hydrology: Regional Studies. (December 2022) 44, 10.1016/j.ejrh.2022.101218.

[19] Roy P. , Pal S. C. , Chakrabortty R. , Chowdhuri I. , and Saha A. , Climate Change and Groundwater Overdraft Impacts on Agricultural Drought in India: Vulnerability Assessment, Food Security Measures and Policy Recommendation, Science of the Total Environment. (November 2022) 849, 10.1016/j.scitotenv.2022.157850. 35934024

[20] Halder S. , RoyChowdhury A. , Kar S. , Ray D. , and Bhandari G. , Critical Watershed Prioritization Through Multi-Criteria Decision-Making Techniques and Geographical Information System Integration for Watershed Management, Sustainability. (April 2024) 16, no. 8, 10.3390/su16083467.

[21] Pande C. , Moharir K. , and Pande R. , Assessment of Morphometric and Hypsometric Study for Watershed Development Using Spatial Technology – A Case Study of Wardha River Basin in Maharashtra, India, International Journal of River Basin Management. (January 2021) 19, no. 1, 43–53, 10.1080/15715124.2018.1505737.

[22] Sar N. , Khan A. , Chatterjee S. , and Das A. , Hydrologic Delineation of Ground Water Potential Zones Using Geospatial Technique for Keleghai River Basin, India, Modeling Earth Systems and Environment. (October 2015) 1, no. 3, 10.1007/s40808-015-0024-3.

[23] Mohammed M. A. A. , Szabó N. P. , and Szűcs P. , Exploring Spatiotemporal Groundwater Flow Patterns in Heterogeneous Systems: A Comprehensive Workflow Combining Multiple Machine Learning Models, Stochastic Environmental Research and Risk Assessment. (November 2025) 39, no. 11, 5029–5048, 10.1007/s00477-025-03063-4.

[24] Godfray G. and Tembo M. M. , Assessment of Ground Water Potentiality in Semi-Arid Area of Central Tanzania. Implication From Geology and Geomorphology of the Dodoman Supergroup, Geology, Ecology, and Landscapes. (April 2024) 8, no. 2, 165–184, 10.1080/24749508.2022.2109833.

[25] Barman J. and Biswas B. , Application of e-TOPSIS for Ground Water Potentiality Zonation Using Morphometric Parameters and Geospatial Technology of Vanvate Lui Basin, Mizoram, NE India, Journal of the Geological Society of India. (October 2022) 98, no. 10, 1385–1394, 10.1007/s12594-022-2186-8.

[26] Pirone M. , Di Maio R. , Forte G. et al., Study of the Groundwater Regime in Unsaturated Slopes Prone to Landslides by Multidisciplinary Investigations: Experimental Study and Numerical Modelling, Engineering Geology. (March 2023) 315, 10.1016/j.enggeo.2023.107045.

[27] Kant C. , Kumar G. , and Meena R. S. , Modeling Morphometric and Geomorphological Parameters of Mountainous River Basin for Water Resource Management Using Remote Sensing and GIS Approach, Modeling Earth Systems and Environment. (June 2023) 9, no. 2, 2151–2163, 10.1007/s40808-022-01614-0.

[28] Das S. and Gupta K. , Morphotectonic Analysis of the Sali River Basin, Bankura District, West Bengal, Arabian Journal of Geosciences. (April 2019) 12, no. 7, 10.1007/s12517-019-4406-0.

[29] Ganie P. A. , Posti R. , Kunal K. et al., Modelling of the Himalayan Mountain River Basin Through Hydro-Morphological and Compound Factor-Based Approaches Using Geoinformatics Tools, Modeling Earth Systems and Environment. (September 2023) 9, no. 3, 3053–3084, 10.1007/s40808-023-01691-9.

[30] Mahala A. , The Significance of Morphometric Analysis to Understand the Hydrological and Morphological Characteristics in Two Different Morpho-Climatic Settings, Applied Water Science. (January 2020) 10, no. 1, 10.1007/s13201-019-1118-2.

[31] Melsse D. W. , Tegegne M. A. , Mekonnen Y. A. , and Bihon Y. T. , Morphometric Analysis for Understanding River Basin Hydrology: A Case of Gelda Watershed, Tana Sub-Basin, Ethiopia, Applied Water Science. (July 2025) 15, no. 7, 10.1007/s13201-025-02526-x.

[32] Taib H. , Hadji R. , and Hamed Y. , Erosion Patterns, Drainage Dynamics, and Their Environmental Implications: A Case Study of the Hammamet Basin Using Advanced Geospatial and Morphometric Analysis, Journal of Umm Al-Qura University for Applied Sciences. (June 2025) 11, no. 2, 235–250, 10.1007/s43994-023-00096-9.

[33] Anteneh Z. L. , Alemu M. M. , Bawoke G. T. , Kehali A. T. , Fenta M. C. , and Desta M. T. , Appraising Groundwater Potential Zones Using Geospatial and Multi-Criteria Decision Analysis (MCDA) Techniques in Andasa-Tul Watershed, Upper Blue Nile Basin, Ethiopia, Review. (March 2021) 10.21203/rs.3.rs-309494/v1.

[34] Shelar R. S. , Nandgude S. B. , Pande C. B. et al., Unlocking the Hidden Potential: Groundwater Zone Mapping Using AHP, Remote Sensing and GIS Techniques, Geomatics, Natural Hazards and Risk. (December 2023) 14, no. 1, 10.1080/19475705.2023.2264458.

[35] Yazdani M. , Zarate P. , Kazimieras Zavadskas E. , and Turskis Z. , A Combined Compromise Solution (Cocoso) Method for Multi-Criteria Decision-Making Problems, Management Decision. (October 2019) 57, no. 9, 2501–2519, 10.1108/MD-05-2017-0458.

[36] Al-Areeq A. M. , Saleh R. A. A. , Ghanim A. A. J. , Ghaleb M. , Al-Areeq N. M. , and Al-Wajih E. , Flood Hazard Assessment in Yemen Using a Novel Hybrid Approach of Grey Wolf and Levenberg Marquardt Optimizers, Geocarto International. (December 2023) 38, no. 1, 10.1080/10106049.2023.2243884.

[37] Debnath J. , Sahariah D. , Mazumdar M. et al., Evaluating Flood Susceptibility in the Brahmaputra River Basin: An Insight Into Asia’s Eastern Himalayan Floodplains Using Machine Learning and Multi-Criteria Decision-Making, Earth Systems and Environment. (December 2023) 7, no. 4, 733–760, 10.1007/s41748-023-00358-w.

[38] Bhattacharya R. K. , Das Chatterjee N. , and Dolui G. , Grain Size Characterization of Instream Sand Deposition in Controlled Environment in River Kangsabati, West Bengal, Modeling Earth Systems and Environment. (September 2016) 2, no. 3, 10.1007/s40808-016-0173-z.

[39] Mahato M. K. and Jana N. C. , Jana N. C. and Singh R. B. , Mapping and Reclamation of Wastelands in Drought-Prone Purulia District of West Bengal, India Using Remote Sensing and GIS, Climate, Environment and Disaster in Developing Countries, 2022, Springer Nature Singapore, Singapore, 189–216, 10.1007/978-981-16-6966-8_10.

[40] Mandal M. , Mandi S. , Modak P. et al., Groundwater Quality Enumeration and Health Risk in the Extended Part of Chhotanagpur Granite Gneiss Complex of India, Environmental Science & Pollution Research. (March 2023) 31, no. 41, 54234–54261, 10.1007/s11356-023-26397-4. 36959399

[41] Chakraborty S. K. , Riverine Ecology Volume 1: Eco-Functionality of the Physical Environment of Rivers, 2021, Springer International Publishing, Cham, 10.1007/978-3-030-53897-2.

[42] Karim S. , Das S. , and DE S. K. , Statistical Analysis of Annual Maximum Flows of a Controlled River: A Study on the Kangsabati River, India, Annals of the National Association of Geographers, India (ATNAGI). (2021) 41, no. 2.

[43] Ghosh A. , Bhattacharjee S. , and Bera B. , Hydro-Geomorphological Mapping of Manbhum-Singhbhum Plateau (Part of Singhbhum Protocontinent, India) for Water Resource Development and Landuse Planning, Journal of the Indian Society of Remote Sensing. (August 2023) 51, no. 8, 1757–1775, 10.1007/s12524-023-01733-w.

[44] Rane N. L. , Achari A. , Saha A. et al., An Integrated GIS, MIF, and TOPSIS Approach for Appraising Electric Vehicle Charging Station Suitability Zones in Mumbai, India, Sustainable Cities and Society. (October 2023) 97, 10.1016/j.scs.2023.104717.

[45] Chakraborty S. , Chatterjee P. , and Das P. P. , Multi-Criteria Decision-Making Methods in Manufacturing Environments: Models and Applications, 2023, 1st edition, Apple Academic Press, New York, 10.1201/9781003377030.

[46] Madhavi S. , Santhosh N. C. , Rajkumar S. , and Praveen R. , Pythagorean Fuzzy Sets-based VIKOR and TOPSIS-Based Multi-Criteria Decision-Making Model for Mitigating Resource Deletion Attacks in WSNs, Journal of Intelligent and Fuzzy Systems. (June 2023) 44, no. 6, 9441–9459, 10.3233/JIFS-224141.

[47] Taherdoost H. and Madanchian M. , An Effective Compromising Ranking Technique for Decision Making, Macro Manag. Public Policies. (May 2023) 5, no. 2, 27–33, 10.30564/mmpp.v5i2.5578.

[48] Krstić M. , Agnusdei G. P. , Tadić S. , Kovač M. , and Miglietta P. P. , A Novel Axial-Distance-Based Aggregated Measurement (ADAM) Method for the Evaluation of Agri-Food circular-economy-based Business Models, Mathematics. (March 2023) 11, no. 6, 10.3390/math11061334.

[49] Pasman H. J. , Rogers W. J. , and Behie S. W. , Selecting a Method/Tool for Risk-Based Decision Making in Complex Situations, Journal of Loss Prevention in the Process Industries. (January 2022) 74, 10.1016/j.jlp.2021.104669.

[50] Chakraborty S. , Applications of the MOORA Method for Decision Making in Manufacturing Environment, The International Journal of Advanced Manufacturing Technology. (2011) 54, 1155–1166, 10.1007/s00170-010-2972-0.

[51] Keshavarz Ghorabaee M. , Zavadskas E. K. , Olfat L. , and Turskis Z. , Multi-Criteria Inventory Classification Using a New Method of Evaluation Based on Distance From Average Solution (EDAS), Informatica. (2015) 26, no. 3, 435–451, 10.3233/INF-2015-1070.

[52] Mengistu T. D. , Chung I.-M. , Kim M.-G. , Chang S. W. , and Lee J. E. , Impacts and Implications of Land Use Land Cover Dynamics on Groundwater Recharge and Surface Runoff in East African Watershed, Water. (June 2022) 14, no. 13, 10.3390/w14132068.

[53] Warku F. , Korme T. , Wedajo G. K. , and Nedow D. , Impacts of Land Use/Cover Change and Climate Variability on Groundwater Recharge for Upper Gibe Watershed, Ethiopia, Sustainable Water Resources Management. (February 2022) 8, no. 1, 10.1007/s40899-021-00588-8.

[54] Choudhari P. P. , Nigam G. K. , Singh S. K. , and Thakur S. , Morphometric Based Prioritization of Watershed for Groundwater Potential of Mula River Basin, Maharashtra, India, Geology, Ecology, and Landscapes. (October 2018) 2, no. 4, 256–267, 10.1080/24749508.2018.1452482.

[55] Singh P. , Kannaujiya A. K. , Deep A. , Singh S. , Mohanty T. , and Prakash K. , Spatio‐Temporal Drought Susceptibility Assessment of Ken River Basin, Central India, and Its Evaluation Through River’s Morphometry, Geological Journal. (February 2023) 58, no. 2, 755–779, 10.1002/gj.4622.

[56] Yáñez‐Morroni G. , Suárez F. , Muñoz J. F. , and Lagos M. S. , Hydrological Modeling of the Silala River Basin. 1. Model Development and Long‐Term Groundwater Recharge Assessment, WIREs Water. (January 2024) 11, no. 1, 10.1002/wat2.1690.

[57] Peach D. and Taylor A. , The Development of a Hydrogeological Conceptual Model of Groundwater and Surface Water Flows in the Silala River Basin, WIREs Water. (January 2024) 11, no. 1, 10.1002/wat2.1676.

[58] Smith A. , Tetzlaff D. , Marx C. , and Soulsby C. , Enhancing Urban Runoff Modelling Using Water Stable Isotopes and Ages in Complex Catchments, Hydrological Processes. (February 2023) 37, no. 2, 10.1002/hyp.14814.

[59] Letz O. , Siebner H. , Avrahamov N. , Egozi R. , Eshel G. , and Dahan O. , The Impact of Geomorphology on Groundwater Recharge in a Semi-Arid Mountainous Area, Journal of Hydrology. (December 2021) 603, 10.1016/j.jhydrol.2021.127029.

[60] Beetle-Moorcroft F. , Shanafield M. , and Singha K. , Exploring Conceptual Models of Infiltration and Groundwater Recharge on an Intermittent River: The Role of Geologic Controls, Journal of Hydrology: Regional Studies. (June 2021) 35, 10.1016/j.ejrh.2021.100814.

[61] Bhakta S. , Barui I. , and Ghosh K. , Identification of Groundwater Potential Zones and Its Relation to Settlement and Agriculture: A Study in Taraphini River Basin, West Bengal, India, Sustainable Water Resources Management. (April 2024) 10, no. 2, 10.1007/s40899-024-01071-w.

[62] Anya B. and Bhuiyan C. , Hydro-Morphometry of a Trans-Himalayan River Basin: Spatial Variance, Inference and Significance, Environmental Challenges. (April 2024) 15, 10.1016/j.envc.2024.100890.

[63] Zanetti F. , Botter G. , and Camporese M. , Stream Network Dynamics of Non‐Perennial Rivers: Insights From Integrated Surface‐Subsurface Hydrological Modeling of Two Virtual Catchments, Water Resources Research. (February 2024) 60, no. 2, 10.1029/2023WR035631.

[64] Bhagwat T. N. , Hegde V. S. , and Shetty A. , Application of Remote Sensing and GIS for Identification of Potential Ground Water Recharge Sites in Semi-Arid Regions of Hard-Rock Terrain, in North Karnataka, South India, Sustainable Water Resources Management. (December 2018) 4, no. 4, 1063–1076, 10.1007/s40899-018-0244-6.

[65] Baah-Kumi B. , Boisramé G. F. S. , Taylor M. H. , and Carroll R. W. H. , Can Reservoir and Groundwater Storage Buffer the Impacts of Climate-Induced Declines in Snowpack Storage for Agriculture and the Environment? Insights From the Walker River Basin, Journal of Hydrology. (February 2024) 630, 10.1016/j.jhydrol.2024.130781.

[66] Poddar I. and Roy R. , Assessing Landslide Susceptibility Mapping in the Sikkim Himalayas Using an Ensemble Machine Learning Approach, Discover Geoscience. (January 2026) 4, no. 1, 10.1007/s44288-025-00375-4.

[67] Zeng X. , Peng X. , Liu T. , Dai Q. , and Chen X. , Runoff Generation Mechanism and Erosion Process at the Rock–Soil Interface of Outcrops With a Concave Surface in a Rocky Desertification Arearunoff Generation Mechanism and Erosion Process at the Rock–Soil Interface of Outcrops With a Concave Surface in a Rocky Desertification Area, SSRN. (2023) 10.2139/ssrn.4487005.

[68] Kang X. , Qi J. , Bourque C. P.-A. , Li S. , Jin C. , and Meng F.-R. , Assessing Watershed-Scale Impacts of Best Management Practices and Elevated Atmospheric Carbon Dioxide Concentrations on Water Yield, Science of the Total Environment. (May 2024) 926, 10.1016/j.scitotenv.2024.171629. 38513862

[69] Dahlke H. E. , LaHue G. T. , Mautner M. R. et al., Managed Aquifer Recharge as a Tool to Enhance Sustainable Groundwater Management in California: Examples from Field and Modeling Studies, Advances in Chemical Pollution, Environmental Management and Protection, 2018, 3, Elsevier, 215–275.

[70] Munir M. U. , Blaurock K. , and Frei S. , Understanding the Vulnerability of Surface–Groundwater Interactions to Climate Change: Insights From a Bavarian Forest Headwater Catchment, Environmental Earth Sciences. (January 2024) 83, no. 1, 10.1007/s12665-023-11314-2.

[71] Zhu B. , Wang X. , and Rioual P. , Multivariate Indications Between Environment and Ground Water Recharge in a Sedimentary Drainage Basin in Northwestern China, Journal of Hydrology. (June 2017) 549, 92–113, 10.1016/j.jhydrol.2017.03.058.

[72] Dar T. , Rai N. , and Bhat A. , Delineation of Potential Groundwater Recharge Zones Using Analytical Hierarchy Process (AHP), Geology, Ecology, and Landscapes. (October 2021) 5, no. 4, 292–307, 10.1080/24749508.2020.1726562.

[73] Zhao L. , Hou R. , Wu F. , and Keesstra S. , Effect of Soil Surface Roughness on Infiltration Water, Ponding and Runoff on Tilled Soils Under Rainfall Simulation Experiments, Soil and Tillage Research. (June 2018) 179, 47–53, 10.1016/j.still.2018.01.009.

[74] Saha S. , Das J. , and Mandal T. , Investigation of the Watershed Hydro-Morphologic Characteristics Through the Morphometric Analysis: A Study on Rayeng Basin in Darjeeling Himalaya, Environmental Challenges. (April 2022) 7, 10.1016/j.envc.2022.100463.

[75] Gajbhiye D. Y. , Shah Z. A. , Konwar M. S. et al., Identification of Potential Groundwater Conservation Sites Using RS & GIS in Raisen District, India, Discov Geosci. (2026) 4, no. 1, 256–2026, 10.1007/s44288-026-00613-3.

[76] Hack J. T. , Stream-Profile Analysis and Stream-Gradient Index, Journal of Research of the U.S. Geological Survey. (1973) 1, no. 4, 421–429.

